# RORα and RORγ expression inversely correlates with human melanoma progression

**DOI:** 10.18632/oncotarget.11211

**Published:** 2016-08-11

**Authors:** Anna A. Brożyna, Wojciech Jóźwicki, Cezary Skobowiat, Anton Jetten, Andrzej T. Slominski

**Affiliations:** ^1^ Department of Tumor Pathology and Pathomorphology, Oncology Centre - Prof. Franciszek Łukaszczyk Memorial Hospital, Bydgoszcz, Poland; ^2^ Department of Tumor Pathology and Pathomorphology, Faculty of Health Sciences, Nicolaus Copernicus University Collegium Medicum in Bydgoszcz, Bydgoszcz, Poland; ^3^ Department of Pharmacodynamics and Molecular Pharmacology, Faculty of Pharmacy, Nicolaus Copernicus University Collegium Medicum in Bydgoszcz, Bydgoszcz, Poland; ^4^ Cell Biology Section, Immunity, Inflammation and Disease Laboratory, National Institute of Environmental Health Sciences, National Institutes of Health, Research Triangle Park, NC, USA; ^5^ Department of Dermatology, Cancer Chemoprevention Program, University of Alabama at Birmingham, AL, USA; ^6^ Comprehensive Cancer Center, Cancer Chemoprevention Program, University of Alabama at Birmingham, AL, USA; ^7^ Pathology and Laboratory Medicine Service, VA Medical Center, Birmingham, AL, USA

**Keywords:** melanoma, RORα, RORγ, melanocytic nevi, vitamin D

## Abstract

The retinoic acid-related orphan receptors (RORs) regulate several physiological and pathological processes, including immune functions, development and cancer. To study the potential role of RORs in melanoma progression, we analysed RORα and RORγ expression in nevi and primary melanomas and non-lesional skin and metastases in relation to melanoma clinico-pathomorphological features. The expression of RORα and RORγ was lower in melanomas than in nevi and decreased during melanoma progression, with lowest levels found in primary melanomas at stages III and IV and in melanoma metastases. Their expression correlated with pathomorphological pTNM parameters being low in aggressive tumors and being high in tumors showing histological markers of good prognosis. Higher nuclear levels of RORα and RORγ and of cytoplasmic RORγ correlated with significantly longer overall and disease free survival time. Highly pigmented melanomas showed significantly lower level of nuclear RORs. This study shows that human melanoma development and aggressiveness is associated with decreased expression of RORα and RORγ, suggesting that RORs could be important in melanoma progression and host responses against the tumor. Furthermore, it suggests that RORα and RORγ might constitute a novel druggable target in anti-melanoma management using tumor suppressor gene therapy restoring their normal functions.

## INTRODUCTION

Melanoma incidence has been increasing during the last few decades with annual increase of incidence ranging from 3% to 7% in white population, especially in older individuals [1–4]. At the same time the mortality rates have stabilized or declined (2-2.6% annually) in younger population, while in older whites 50 and older mortality rates have been increased (from 0.2 to 1.1 % per year [5]). The improved survival results mostly from diagnosis of early-stage melanomas [1]. The most efficient treatment mode is surgical excision, but it is limited to the localized disease (stage I and II). The efficacy of conventional therapies as surgery, chemotherapy, or radiotherapy for the treatment of advanced melanomas, is limited and related to adverse effects. In recent years new therapeutic approaches, based on modulation of immune responses and targeting molecular pathways, have been approved for the treatment of metastatic melanoma [2]. These new anti-melanoma strategies are related to less adverse effects and improved disease free survival. However, they also poses some limitations [1]. Therefore, studies on new regulatory targets and effective approaches in treatment of advanced melanomas are fully justified.

The human nuclear receptor (NR) superfamily is a highly conserved family of transcription factors, which includes receptors for steroid hormones, vitamin D hydroxyderivatives, retinoids, thyroid hormones, and lipids and oxysterols [6–8]. The disturbances in NRs expression are observed in many pathological conditions. Previously we demonstrated that expression of nuclear receptor for vitamin D (VDR) in melanomas decreased with tumor progression. In addition, lack of VDR correlated with shorter overall survival (OS) and disease-free survival (DFS) time [9, 10]. The retinoic acid receptor-related orphan receptor (ROR) family consists of RORα (NR1F1), RORβ (NR1F2), and RORγ (NR1F3), members of the NR superfamily [12, 13]. Their activity is regulated by endogenous ligands, including 7-dehydrocholesterol, cholesterol and its derivatives [7, 11–13]. Most recently we demonstrated that RORα and RORγ are expressed in normal and pathological human skin and that hydroxyderivatives of vitamin D can act as reverse agonists of these receptors [14]. These novel vitamin D3 hydroxyderivatives are produced ex-vivo by adrenal and placenta fragments as well as epidermal keratinocytes [15], and are detectable in human epidermis and serum [16]. RORs demonstrate a typical NR modular structure, containing a highly conserved DNA binding domain and a less well conserved putative ligand binding domain. They regulate transcription by binding to ROR-responsive elements (ROREs) in the regulatory regions of target genes [6]. RORs are involved in the regulation of diverse, fundamental physiological processes related to cell development, growth, differentiation, apoptosis and immune functions [6, 17–19]. They show a tissue and/or cell type specific pattern of expression [20–30]. RORα stabilizes p53 and enhances DNA damage-induced apoptosis through p53 [31]. It inhibits angiogenesis [32, 33] and inhibits the NF-κB pathway [21, 34]. RORα can also stimulate keratinocytes differentiation [35] and is expressed in all skin cell types [36, 14]. The human *RORγ* gene generates two isoforms, which exhibit a distinct tissue-specific expression pattern and play important role in immune regulation (reviewed in [6, 18]. RORγt is essential for the polarization of CD4+ T helper cells into Th17 cells [37–39] and is involved in (auto)immune responses [40].

Biological functions regulated by RORs are important for the oncogenesis and tumor progression [41, 29, 42, 43]. Lack of RORγ is related to a high incidence of thymic lymphomas in mice [19, 29]. Although a tumor suppressive role of RORs was demonstrated in experimental *in vivo* and *in vitro* models [41, 29, 42–44], description of RORs expression in relation to human tumor progression and relation to clinico-pathological features has been superficial, at best. In addition, there is a lack of information on the role of RORα and RORγ in skin tumors. Although we detected a strong nuclear expression of both RORα and RORγ in human normal skin, ROR expression showed a very heterogeneous pattern in four invasive melanoma samples [14]. Therefore, we decided to examine RORα/γ expression in benign (nevi) and malignant (melanomas) melanocytic tumors and to correlate their relationship with tumor progression and association with pathomorphological and clinical features including disease free and overall survival, to obtain patient relevant information on their role in human melanomagenesis and melanoma progression.

## RESULTS

### RORα and RORγ expression in human skin and melanocytic lesions

**Figure 1:**
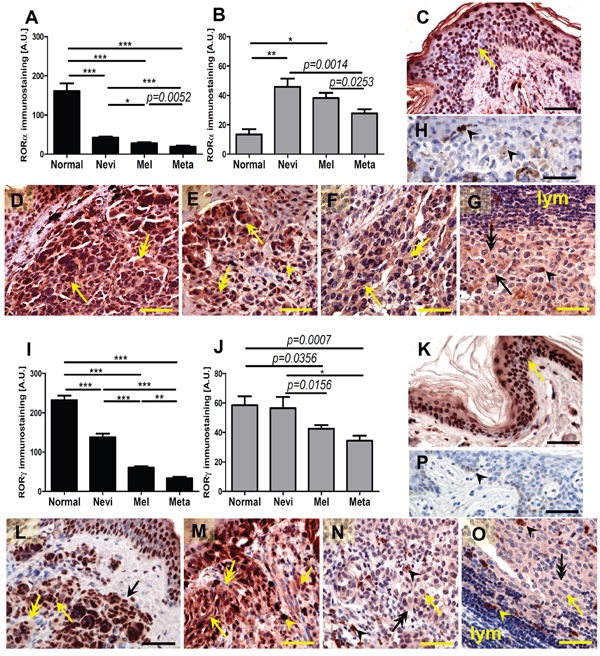
The mean level of nuclear (A, I) and cytoplasmic (B, J) RORα (A, B) and RORγ (I, J) in keratinocytes of normal skin, melanocytic cells of nevi, primary melanomas (MM) and metastases (meta) Statistically significant differences are denoted with *P* values as determined by Student's *t*-test and with asterisks by ANOVA (*P<0.05, **P<0.01 and ***P<0.001). Representative RORα (**C-H)** and RORγ (**K-P)** immunostaining of normal skin (**C, K**), melanocytic nevi (**D, L**), early-stage melanomas (**E, M**), advanced melanomas (**F, N**) and lymph node metastases (**G, O**) Negative controls are presented at H and P panels. Arrows indicate nuclear staining in melanomas, arrow heads - melanin, double arrows - cytoplasmic staining, lymph - lymphocytes, scale bars - 50μm.

Expression of RORα and RORγ protein was detected in all analyzed normal and pathological samples (Figure 1A–1P). RORα and γ showed both nuclear and cytoplasmic localization; however, the pattern of immunostaining was different for both receptors (Figure 1C–1G, 1K–1O). In keratinocytes of normal skin RORα showed a predominantly nuclear localization, while in cells of melanocytic lesions (nevi, primary melanomas and melanoma metastases) both cytoplasmic and nuclear expression was equally observed. Detailed analysis revealed significantly higher expression of RORα in nuclei of normal keratinocytes in comparison to melanocytic cells of pathological samples (Figure 1A), while the cytoplasmic RORα expression in keratinocytes of normal skin was significantly lower than that in cell of melanocytic nevi and primary melanomas (Figure 1B). In melanocytic tumors, both nuclear and cytoplasmic RORα gradually decreased with the progression of melanocytic lesions and melanomas (Figure 1B).

RORγ showed significantly higher expression in both nuclei and cytoplasm of keratinocytes of normal skin in comparison to cells of melanocytic tumors, but exhibited similar cytoplasmic expression vs cells of melanocytic nevi (Figure 1I–1J). There was also a gradual decrease of RORγ expression during progression of melanocytic tumors from nevus to primary melanoma with lowest expression seen in melanoma metastases. These differences were most pronounced for the nuclear localization.

Comparison of matched primary melanomas and its metastases revealed that in 24 out of 36 cases, nuclear RORγ was higher in the primary tumors. Wilcoxon matched-pairs signed rank test for 36 patients showed significant reduction of nuclear RORγ in metastases when compared to primary lesions (p = 0.028).

**Figure 2:**
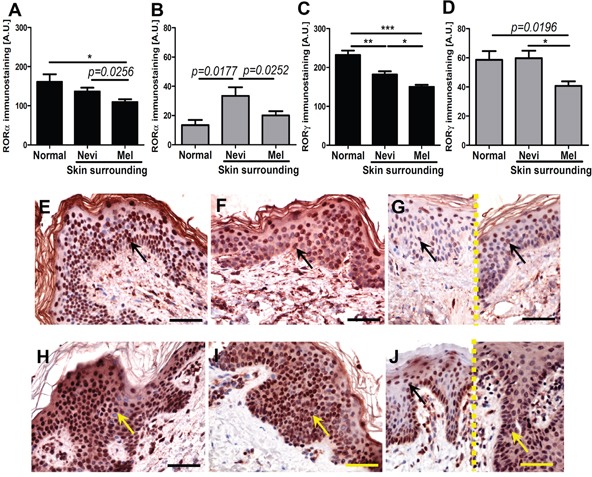
The mean level of nuclear (A, C) and cytoplasmic (B, D) RORα (A, B) and RORγ (C, D) in keratinocytes of normal skin and keratinocytes of skin surrounding melanocytic nevi and melanomas Statistically significant differences are denoted with *P* values as determined by Student's *t*-test and with asterisks by ANOVA (*P<0.05, **P<0.01 and ***P<0.001). Representative RORα (**E-G**) and RORγ (**H-J**) immunostaining of normal skin (**E, H**), skin surrounding melanocytic nevi (**F, I**) and skin surrounding melanomas (**G, J** different cases, separated with dashed line). Arrows indicate nuclear staining in melanomas, scale bars - 50μm.

Similar trends for RORα and RORγ expression were also observed for keratinocytes of normal skin vs keratinocytes of skin surrounding nevi and melanomas (Figure 2A–2J). Expression of both nuclear and cytoplasmic RORγ in keratinocytes of skin surrounding nevi and melanomas gradually decreased being the lowest in skin surrounding melanomas (Figure 2C–2D, 2H–2J). For RORα, this gradual decrease from keratinocytes of normal to perilesional skin was found only for nuclear localization (Figure 2A, 2E–2G). Cytoplasmic RORα was elevated in keratinocytes of skin surrounding nevi in comparison to keratinocytes of normal skin or keratinocytes of skin surrounding melanomas (Figure 2B, 2E–2G).

**Figure 3:**
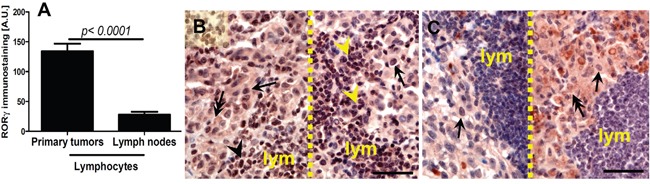
Comparison of RORγ in lymphocytes in primary melanomas and metastatic lymph nodes (A) Statistically significant differences are denoted with *P* values as determined by Student's *t*-test. Representative RORγ immunostaining of lymphocytes (lym) in primary melanoma (**B**, two different cases, separated with dashed line) and metastatic lymph node (**C,** two different cases, separated with dashed line). Arrows indicate nuclear staining in melanomas, arrow heads - staining in lymphocytes, double arrows – cytoplasmic staining in melanomas, scale bars - 50μm.

We also observed significantly higher RORγ expression in lymphocytes infiltrating primary melanomas localized to the skin in comparison to lymphocytes surrounding metastatic melanomas in the lymph nodes (Figure 3A–3C). There was no relationship between levels of RORα and RORγ expression and anatomic localization of the lesion.

### RORα and RORγ expression in tissue samples is affected by melanogenesis

**Figure 4:**
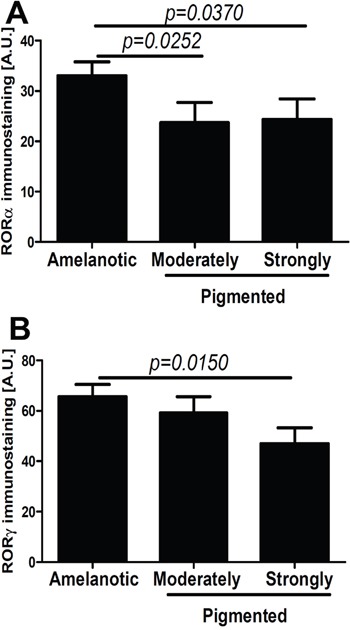
Comparison of nuclear RORα (A) and RORγ (B) in amelanotic, moderately and strongly pigmented primary melanomas Statistically significant differences are denoted with *P* values as determined by Student's *t*-test.

We also tested the correlation between melanin pigmentation and RORα and RORγ expression in primary melanoma tumors (Figure 4A–4B), and have found that its nuclear immunoreactivity was inversely correlated with high melanin content (r=−0.2920, P=0.0052 and r=−0.2109, p=0.0399, respectively). The melanomas with high melanin content showed significantly lower levels of nuclear RORα and RORγ than amelanotic and moderately pigmented melanomas (Figure 4A–4B). Moreover, these relationships were more pronounced for RORα expression and vertical growth phase of melanomas (data not shown). There was no correlation for cytoplasmic RORα and RORγexpressionand melanin content.

**Figure 5:**
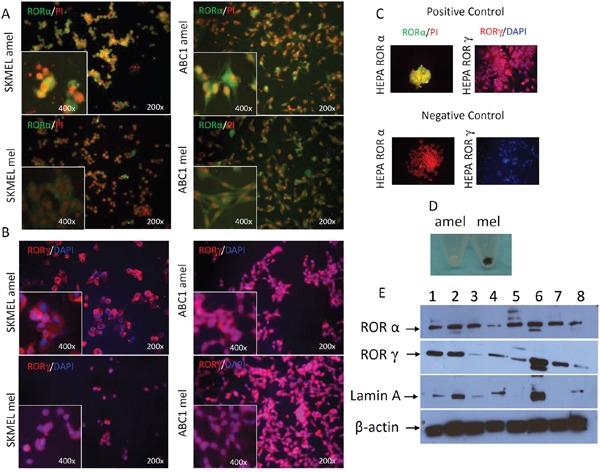
Changes in RORα and RORγ expression pattern in cultured melanoma cells after induction of melanogenesis F-ICC, RORα expression (green) in SKMEL amelanotic (amel), SKMEL melanotic (mel), ABC1 amelanotic (amel) and ABC1 melanotic (mel); PI was used to counterstain nuclei (**A**). F-ICC, RORγ expression (red) in SKMEL amel, SKMEL mel, ABC1 amel, ABC1 mel; DAPI used to counterstain nuclei (**B**). Examples of positive and negative controls (**C**). The differences in pigmentation between melanotic (mel) and amelanotic (amel) SKMEL cells (**D**). WB analysis of RORα and RORγ expression (**E**). Proteins were extracted separately from cytoplasm (1, 3, 5, 7) and nucleus (2, 4, 6, 8) of human SKMEL (amelanotic 1-2, melanotic 3-4), hamster AbC1 (melanotic 5-6) melanomas and HEPA cells as a positive control (7-8) and stained with antibodies against RORα, RORγ. Arrows in two upper panels show proteins with an expected molecular mass of ~67 kDa for RORα and of ~63 kD for RORγ. Two lower panels show, respectively, stains for Lamin A (nuclear marker) and β-actin (expressed in both compartments), which were used as loading controls (**E**).

We also tested RORα and RORγ expression in cultured melanoma cells using immunofluorescence (IF) and Western Blot (WB) techniques (Figure 5). RORα and RORγ antigens were detected in both cytoplasmic and nuclear compartments of melanoma cells (Figure 5A, 5B, 5E), and in positive controls consisting of HEPA cells overexpressing RORα or RORγ, while being absent in negative controls (Figure 5C). The phenotype of amelanotic and melanotic cells is presented in Figure 5D. The IF signals were higher in amelanotic cells vs melanotic melanoma cells (Figure 5A, 5B). The WB confirmed expression of RORα and RORγ in both nuclear and cytoplasmic fractions of human SKMEL and hamster AbC1 melanomas, as well as control HEPA cells overexpressing RORs (Figure 5E). WB analysis also showed a decrease (relative to Lamin A as control) in RORα or RORγ expression in melanized human melanoma nuclear extracts in comparison to amelanotic cells by 4% and 10%, respectively (Figure 5E).

### RORα and RORγ expression correlates to melanoma advancement and melanoma prognostic markers (proliferation, ulceration, TILs, histological type)

**Figure 6:**
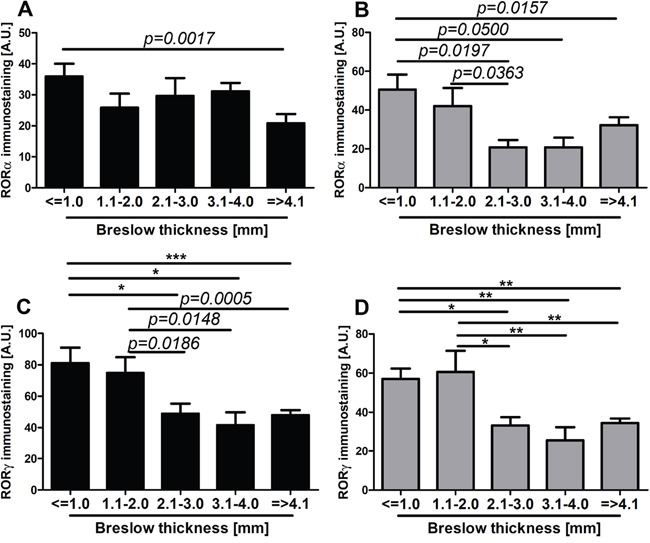
The mean level of nuclear (A, C) and cytoplasmic (B, D) RORα (A, B) and RORγ (C, D) in primary melanomas stratified according to the Breslow thickness Statistically significant differences are denoted with *P* values as determined by Student's *t*-test and with asterisks by ANOVA (*P<0.05, **P<0.01 and ***P<0.001).

**Figure 7:**
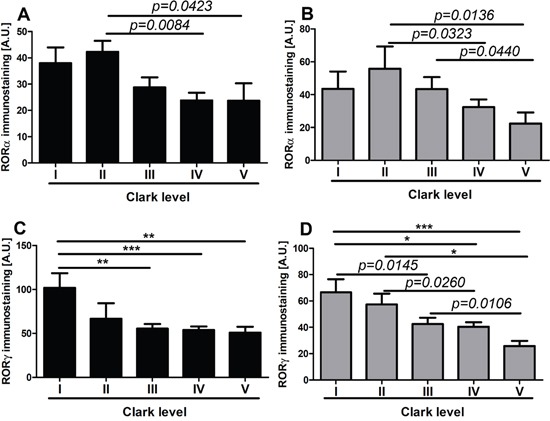
The mean level of nuclear (A, C) and cytoplasmic (B, D) RORα (A, B) and RORγ (C, D) in primary melanomas stratified according to the Clark level Statistically significant differences are denoted with *P* values as determined by Student's *t*-test and with asterisks by ANOVA (*P<0.05, **P<0.01 and ***P<0.001).

RORα and RORγ expression inversely correlated with melanoma progression, aggressiveness and prognostic markers with less advanced melanomas exhibiting higher expression of RORα and RORγ (Figure 6–Figure 11; Table 1). The detailed analysis of RORα and RORγ in primary melanomas stratified according to Clark and Breslow levels revealed significant negative correlation between expression of RORα and RORγ and progression of melanoma (Table 1). Melanomas at less advanced stages (Clark levels I and II and Breslow thickness ≤2 mm) (Figure 1E–1F, 1M–1N, Figure 6, Figure 7) showed highest level of both nuclear and cytoplasmic expression of RORα and RORγ. Moreover, a significant decrease of cytoplasmic and nuclear expression of RORγ was seen in melanoma cells growing in the reticular dermis in comparison to the cells localized in the papillary dermis (Figure 12A). Analysis of mean staining intensity of RORγ showed similar trend (Figure 12B), although in some melanoma cases focally nuclear RORγ was stronger in reticular than papillary dermis.

**Table 1:** Correlation of RORα and RORγ levels with pathomorphological features

	Nuclear RORα	Cytoplasmic RORα	Nuclear RORγ	Cytoplasmic RORγ
**Clark level (n=79)**	r=−0.1952 p=0.0424	r=−0.2521 p=0.0125	r=−0.3238 p=0.0020	r=−0.4065 p=0.0001
**Breslows thickeness (n=79)**	NS	r=−0.1918 p=0.0485	r=−0.2777 p=0.0076	r=−0.3152 p=0.0028
**pT (n=79)**	r=−0.3364 p=0.0012	r=−0.2940 p=0.0043	r=−0.3696 p=0.0005	r=−0.3884 p=0.0002
**pN (n=79)**	r=−0.2810 p=0.0066	r=−0.2470 p=0.0152	r=−0.3428 p=0.0011	r=−0.3442 p=0.0011
Overall stage* (n=79)	r=−0.3134 p=0.0035	r=−0.3094 p=0.0039	r=−0.4977 p< 0.0001	r=−0.3857 p=0.0003
Overall stage** (n=79)	r=−0.3260 p=0.0017	r=−0.2868 p=0.0052	r=−0.4897 p<0.0001	r=−0.4410 p<0.0001
**TILs (n=76)#**	r=0.3589 p=0.0008	r=0.2213 p=0.0282	r=0.2623 p=0.0106	NS
**Ulceration (n=78)##**	r=−0.3725 p=0.0004	r=−0.1635 p=0.0776	r=−0.4302 p< 0.0001	r=−0.3318 p=0.0016
**Proliferative index (Ki-67 immunostaining) (n=74)###**	NS	NS	NS	r=−0.2159 p=0.0333

* assessed at the time of diagnosis

** assessed during follow-up

NS- not statistically significant

# missing data for 3 cases

## missing data for 1 case

### missing data for 5 cases

**Figure 8:**
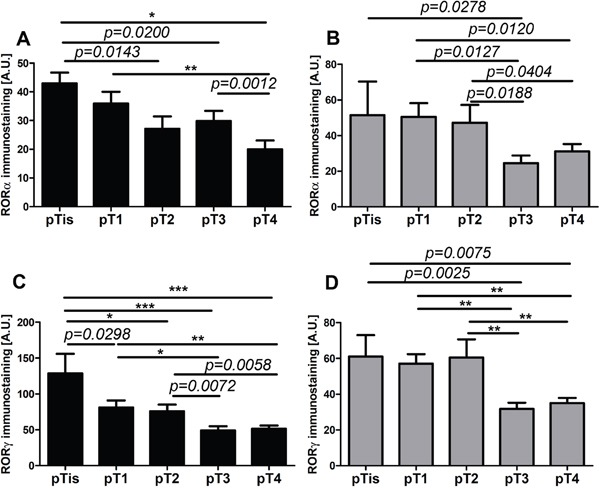
The mean level of nuclear (A, C) and cytoplasmic (B, D) RORα (A, B) and RORγ (C, D) in primary melanomas stratified according to the pT Statistically significant differences are denoted with *p* values as determined by Student's *t*-test and with asterisks by ANOVA (*P<0.05, **P<0.01 and ***P<0.001).

**Figure 9:**
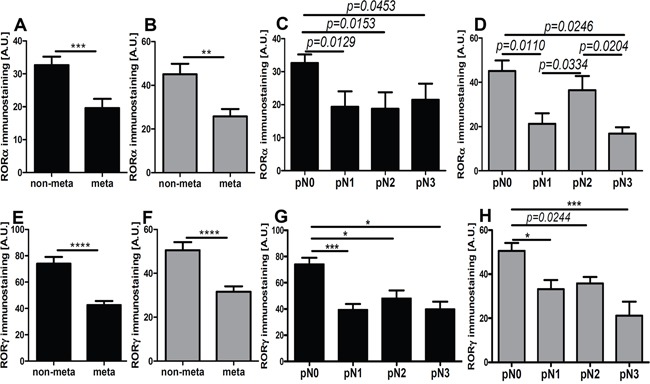
The mean level of nuclear (A, C, E, G) and cytoplasmic (B, D, F, H) RORα (A-D) and RORγ (E-H) in primary melanomas stratified according to the presence of metastases presence and pN status Statistically significant differences are denoted with *P* values as determined by Student's *t*-test and with asterisks by ANOVA (*P<0.05, and ***P<0.001).

**Figure 10:**
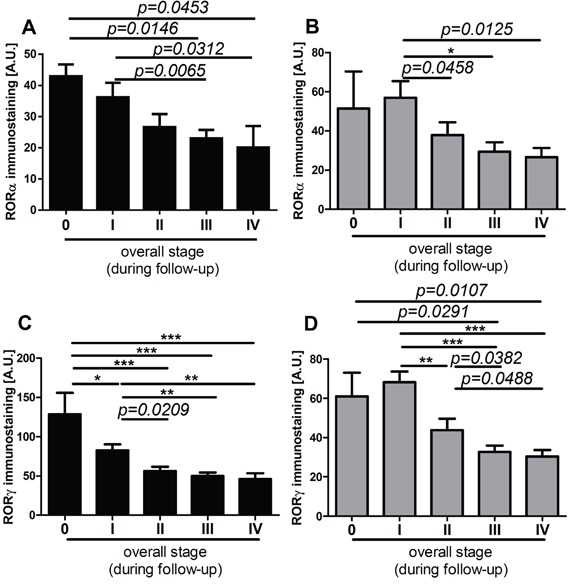
The mean level of nuclear (A, C) and cytoplasmic (B, D) RORα (A, B) and RORγ (C, D) in primary melanomas stratified according to the disease stage assessed during follow-up of patients Statistically significant differences are denoted with *P* values as determined by Student's *t*-test and with asterisks by ANOVA (*P<0.05, **P<0.01 and ***P<0.001).

**Figure 11:**
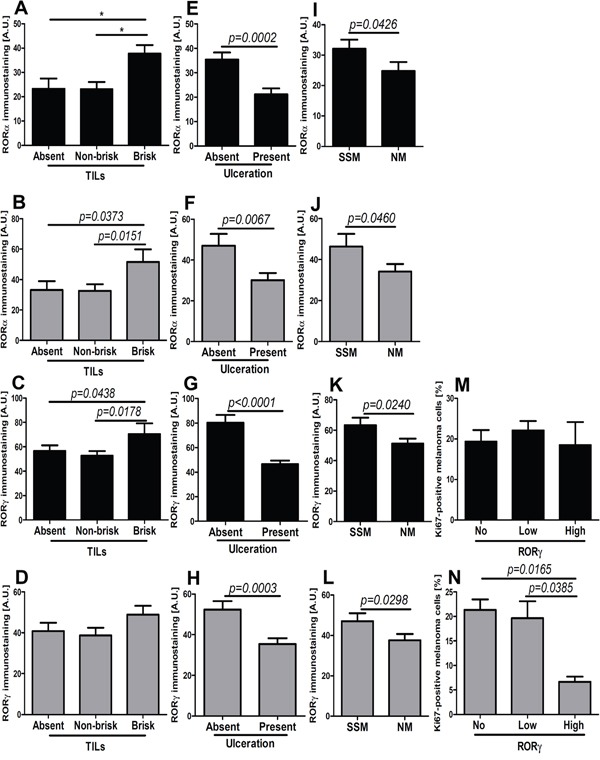
Comparison of mean level of nuclear (black bars) and cytoplasmic (gray bars) RORα (A, B, E, F, I, J) and RORγ (C, D, G, H, K-N) in primary melanomas without and with tumor-infiltrating lymphocytes (TILs) (A-D), without and with ulceration (E-H), stratified according histological type (I-L, SSM - superficial spreading melanomas; NM - nodular melanomas) and Ki-67 immunostaining (M, N) Statistically significant differences are denoted with *P* values as determined by Student's *t*-test and with asterisks by ANOVA (*P<0.05, **P<0.01 and ***P<0.001).

**Figure 12:**
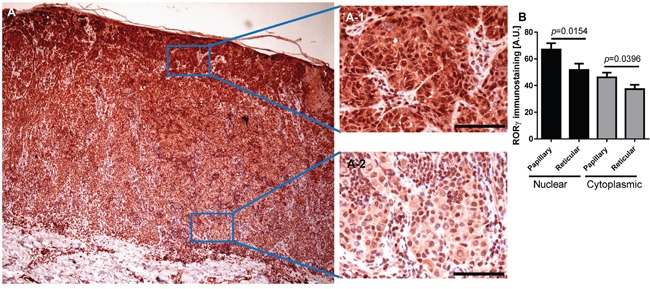
Representative immunostaining of RORγ in melanoma RORγ expression gradually decreased with increasing melanoma depth **(A)** A-1 and A-2 represent fragments indicated by squares in Figure (A) Scale bars - 100μm. **(B)** Mean nuclear (black bars) and cytoplasmic (gray bars) RORγ immunostaining in melanoma cells within the papillary and reticular dermis. Statistically significant differences are denoted with *P* values calculated with t-test.

We found a similar correlation when primary melanomas were classified according to AJCC pTNM stage. The negative correlation was observed between pT stage and pN and nuclear and cytoplasmic level of RORα and RORγ (Table 1). The comprehensive analysis of mean level of RORα and RORγ revealed that less advanced melanomas (pTis, pT1, and pT2) showed significantly higher RORα and RORγ expression than more advanced melanomas (pT3 and pT4) (Figure 8A–8D).

We also analyzed in detail relative expression of RORs in relation to the ability to develop metastases and found that primary melanomas that metastasized showed reduced nuclear and cytoplasmic expression of RORα and RORγ in comparison to non-metastasizing melanomas (Figure 9A–9B, 9E–9F). When primary melanomas were subgrouped according to pN stage, significant reduction of both nuclear and cytoplasmic of analyzed markers was observed in stages pN1, pN2, and pN3 (advanced stages) vs pN0 (Figure 9C–9D, 9G–9H).

Lastly, we analyzed RORα and RORγ expression in relation to overall stage of the diseases and found significant negative correlation between overall stage and nuclear and cytoplasmic levels of RORα and RORγ (Table 1). Furthermore, analysis of primary melanomas stratified according overall stage demonstrated that melanomas at very early stages of development (stage 0 and 1) had significantly higher both nuclear and cytoplasmic RORα and RORγ levels than primary melanomas at stage II and primary melanomas with regional and distant metastases (stages III and IV). This trend was observed for melanomas at stages assessed at the time of diagnosis (data not shown) and during progression of the disease, as assesses during follow-up (Figure 10A–10D).

Next, we analyzed the correlation between RORα and RORγ levels in primary melanomas and the presence of tumor-infiltrating lymphocytes (TILs), proliferation activity and histological type. Both nuclear and cytoplasmic RORα correlated positively with TILs, but for RORγ such correlation was found only for the nuclear localization (Table 1). Specifically, significantly higher nuclear and cytoplasmic RORα and nuclear RORγ levels were found in melanomas with brisk TILs (Figure 11A–11D). The presence of ulceration was correlated with significant reduction of both nuclear and cytoplasmic stains for RORα and RORγ (Figure 11E–11H; Table 1). Similarly, more aggressive nodular type melanomas (NM) showed substantial decrease of RORα and RORγ expression (both nuclear and cytoplasmic) in comparison to superficial spreading (SSM) melanomas (Figure 11I–11L). Proliferation activity, as assessed by Ki-67 immunocytochemistry, had no correlation with cytoplasmic and nuclear RORα levels (Table 1) or nuclear RORγ expression (Figure 11M), except that higher cytoplasmic RORγ staining was observed in melanomas with lower proliferation levels (Figure 11N). There was no relationship between RORα and RORγ levels and mitotic rate calculated as the mean number of mitoses per mm^2^ (data not shown).

### RORα and RORγ expression affects OS and DFS of melanoma patients

**Figure 13:**
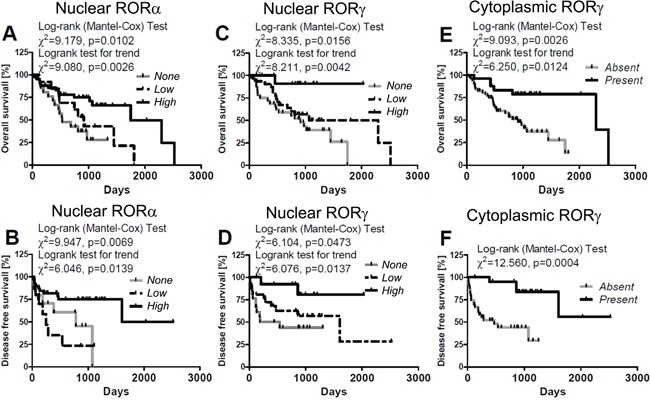
Correlation of overall survival (A, C, E) and disease-free (B, D, F) survival time with relative expression of nuclear RORα (A, B) and RORγ (C, D) and cytoplasmic RORγ in primary melanomas (E, F) Melanomas were classified as “high”, “low” and “none” according to RORs SQ-score; for details see Material and method section: 4.1.3. Evaluation of RORα and RORγ immunostained sections.

Importantly, there was a correlation between the expression of RORα and RORγ in primary melanomas and OS and DFS in cohort of melanoma patients included into this study. Nuclear RORα and RORγ affected both OS and DFS (Figure 13A–13F, Table 2). High nuclear level of RORα and RORγ correlated with significantly longer OS (χ^2^ = 9.179, P = 0.0102 and χ^2^ = 8.335, P = 0.0156, respectively) and DFS (χ^2^ = 9.947, P = 0.0069 and χ^2^ = 6.104, P=0.0473, respectively).

**Table 2:** Comparison of overall survival and disease free survival time in patients with RORα and RORγ expression in primary melanomas and metastases (Log-rank Mantel-Cox Test)

Expression of:	Total cases (n)	Deaths (n/%)	Median/mean overall survival (days)	Statistically significant p values	Metastases (n/%)	Median/mean disease free survival (days)	Statistically significant p values
nuclear RORα							
None	22	14/63.6	618.3/530.0		13/59.1	288.3/69.00	
Low	14	9/64.3	843.1/876.0	P>0.05*	11/78.6	275.4/127.0	P>0.05*
High	43	16/37.2	896.1/899.0	P=0.0064*	15/34.9	689.7/597.0	P=0.0302*
				P>0.05^^^			P=0.0030^^^
cytoplasmic RORα							
None	14	8/57.1	720.7/876.0		7/50.0	470.3/263.0	
Low	18	11/61.1	813.4/819.0	P>0.05*	12/66.7	338.7/152.0	P>0.05*
High	47	20/42.6	841.4/825.0	P>0.05*	21/44.7	580.9/429.0	P>0.05*
				P>0.05^^^			P>0.05^^^
nuclear RORγ							
None	32	20/62.5	733.9/779.0		19/59.4	298.3/114.0	
Low	33	18/55.5	872.4/829.0	P>0.05*	18/55.5	539.2/284.0	P>0.05*
High	14	1/7.1	860.7/877.0	P=0.0053*	2/14.3	842.7/877.0	P=0.0091*
				P=0.0373^^^			P=0.0337^^^
cytoplasmic RORγ							
None	53	30/56.6	713.9/714.0		32/60.4	340.5/127.0	
Low	23	8/34.8	1007.0/940.0	P=0.0080*	7/30.4	818.7/916.0	P=0.0006*
High	3	0/0.0	1101.0/1204.0	P=0.0231*	1/33.3	1101.0/1204.0	P=0.0405*
				P>0.05^^^			P>0.05^^^

* versus No ROR

^ versus Low ROR

Detailed analysis of melanoma patients stratified according to the level of nuclear RORα expression revealed longer OS in patients with high (median survival 896.1 days) expression of the receptor in comparison to those that lacked nuclear stain (median survival 618.3 days) (χ^2^ = 7.420, P = 0.0064, Figure 13A, Table 2). Similar differences were observed also in DFS between cases without RORα and with high nuclear RORα (median disease free survival 288.3 days vs 689.7 days; χ^2^ = 4.697, P=0.0302, Figure 13B, Table 2) and cases with low vs high RORα expression (median disease free survival 275.4 days vs 689.7 days; χ^2^ = 8.835, P = 0.0030, Figure 13B, Table 2).

Similar differences were seen in OS curves of patients with melanomas lacking nuclear RORγ vs those with high nuclear RORγ (χ^2^ = 7.768, P=0.0053) or melanomas with low vs high nuclear RORγ stains (χ^2^ = 4.336, P = 0.0373) (Figure 13C, Table 2). Also DFS curves showed significant differences for patients with melanomas expressing high RORγ vs melanomas negative for RORγ (χ^2^= 6.797, P = 0.0091) and melanomas with low nuclear RORγ (χ^2^ = 4.510, P = 0.0337; Figure 13D, Table 2).

Because of low number of cases with cytoplasmic stain for RORγ (n=3), the patients were stratified into two groups: with and without RORγ and statistically significant differences were found for OS and DFS curves in these groups (χ^2^ = 9.093, P = 0.0026 for OS and χ^2^ = 12.560, P = 0.0004 for DFS) (Figure 13E, 13F, Table 2). Interestingly, no correlation was found for OS and DFS in patients stratified according to cytoplasmic expression of RORα (Table 2). The t-test analysis confirmed Mantel-Cox test both for OS and DFS (data not shown).

Similarly, a significant better prognosis was also observed for melanoma patients with nuclear RORα immunolocalization and with nuclear and cytoplasmic RORγ immunolocalization after adjustment for Breslow thickness (Table 3). After adjustment for overall stage these relationship were significant for DFS both for nuclear RORα imunolocalization and nuclear and cytoplasmic RORγ immunolocalization (Table 3) and for OS only for nuclear RORγ (χ2=22,396, p<0.0001, b=−0,391, SE=0,343, p=0,0357, Exp(b)= 0,676, 95% CI of Exp(b)=1,4475 to 3,0928).

**Table 3:** Survival analysis using Cox proportional-hazards regression analysis of risk factors in relation to RORs immunostaining in human melanomas adjusted for Breslow thickness (A) and overall stage (B)

A							
	χ2	P value	b	SE	P value	Exp(b)	95% CI
**Overall survival**							
**Nuclear RORα**	12.557	0.002	−0.804	0.357	0.024	0.448	0.222 to 0.902
**Nuclear RORγ**	13.859	0.001	−0.864	0.349	0.013	0.421	0.212 to 0.835
**Cytoplasmic RORγ**	19.810	<0.0001	−1.481	0.492	0.003	0.227	0.087 to 0.596
**Disease-free survival**							
**Nuclear RORα**	7.349	0.0254	−0.660	0.439	0.132	0.517	0.219 to 1.221
**Nuclear RORγ**	8.586	0.0137	−1.132	0.438	0.010	0.322	0.136 to 0.762
**Cytoplasmic RORγ**	17.831	0.0001	−2.111	0.653	0.001	0.121	0.034 to 0.436
**B**							
**Disease-free survival**							
**Nuclear RORα**	44.427	<0.0001	−0.954	0.453	0.035	0.385	0.159 to 0.937
**Nuclear RORγ**	44.757	<0.0001	−0.917	0.439	0.037	0.400	0.169 to 0.945
**Cytoplasmic RORγ**	46.838	<0.0001	−1.543	0.683	0.024	0.214	0.056 to 0.816

b – regression coefficient; SE - standard error; Exp(b) – relative risk; 95% CI - 95% confidence interval for Exp(b); Lack of RORs (= 0) versus RORs present (= 1).

## DISCUSSION

This study represents the first comprehensive analysis of RORα and RORγ in human melanocytic tumors in relation to several clinico-pathomorphological features associated with various stages of melanoma progression. Specifically, nuclear expression of RORα and RORγ was lower in cells of melanomas in comparison to melanocytic nevi and keratinocytes of normal skin and decreased during progression of melanoma, with lowest levels found in primary melanomas at stages III and IV and in melanoma metastases. Concerning cytoplasmic expression, RORα levels were lower in keratinocytes of normal skin vs cells of melanocytic nevus and primary melanomas; however, a decrease of its expression was seen in primary and metastatic melanomas vs melanocytic nevus. Cytoplasmic RORγ was higher in keratinocytes of normal skin and cells of melanocytic nevi vs cells of localized and metastatic melanomas. Moreover, RORα and RORγ expression correlated with pathomorphological pTNM parameters. Higher RORs levels positively correlated with markers of good prognosis such as SSM vs NMM histological type, presence of brisk TILs, absence of ulceration and low proliferation activity.

Until recently, there was a shortage of information on RORs expression in resident cells of human skin, except of few papers analyzing expression of RORα [35, 45, 36]. In human keratinocytes RORα promotes epidermal differentiation and expression of structural proteins involved in lipid barrier formation [35, 45]. Importantly, the same authors [35] found reduced RORα expression in skin squamous cell carcinoma tumors and squamous cell lines, indicating reverse correlation between epidermal cancer and RORα expression. Previously, we reported RORα and RORγ expression in cells of human normal skin, including epithelial cells of the epidermis, hair follicle, sebaceous and sweat glands, dermal fibroblasts, immune cells and cultured melanocytes and melanoma lines [14]. Since initial studies have shown that the expression of RORα and RORγ in melanomas was aberrant and heterogeneous, further detailed examination is presented in this study.

The decreased expression of RORα and RORγ during progression of melanocytic lesions and the significant reduction of their expression in most advanced primary melanomas (Breslow thickness >2mm, Clark level III-V, pT3-4, pN1-3, overall stage II-IV) and melanoma metastases suggest that reduced or lack of RORα and RORγ expression or defects in the ROR pathways could promote tumor progression. Lack or significant reduction of RORα and RORγ expression in primary melanomas and melanoma metastases would be consistent with this concept. As mentioned above, RORα and RORγ exhibit, inter alia, anti-tumor effects and disturbances in their functions and expression can contribute to a development and progression of malignant lesions. A reduced level of RORα has been associated with several other cancers, including hepatocellular, breast, lung, colon, cervical, ovary and prostate cancers [46, 47, 44]. In mouse models activation of RORα inhibited growth of androgen-independent prostate cancer cells [41]. Increased RORα expression suppressed also the aggressive phenotype in breast cancer models, including reduced colony size, inhibited cell proliferation, invasion and migration [48]. Moreover, reduced RORα levels correlated with shorter OST in patients with breast and hepatocellular cancers [46, 48]. The crucial role of RORs as anti-tumor factors is further supported by studies showing a negative correlation of RORα and RORγ expression with pathological grade, increased incidence of tumor recurrence, vascular invasion, clinical outcome, including distance metastasis free survival and OST [46, 49, 50]. Correspondingly, in the present study the significant reduced RORα and RORγ expression in melanomas correlated to poorer clinical outcome, as determined by overall and disease-free survival time. Furthermore, in melanomas lower level of RORα and RORγ was associated with markers of poor prognosis such as absent or non-brisk TILs, nodular histological type, presence of ulceration and higher proliferation index, indicating that a reduced level of RORα and RORγ promotes a more aggressive melanoma phenotype. Together, these results suggest that RORs are very important in oncogenesis and tumor progression. Positive correlation between decreased RORα and RORγ expression with progression of melanomas suggests the potential usefulness of RORα and RORγ as new, immunohistochemical marker in melanoma diagnosis, predicting future behavior of the lesion. Additionally, the shorter overall and disease-free survival time when RORα and RORγ were absent suggests that their presence or absence could serve as a prediction marker of clinical outcome.

The reduced RORα and RORγ levels were also observed in melanized melanomas. This was further substantiated by testing of melanoma lines in which induction of melanogenesis by L-tyrosine [51, 52] was accompanied by reduced levels of nuclear RORα and RORγ. This is consistent with our previous observation showing the reduction of OS and DFS of patients with a pigmented melanomas at stage 3 and 4 [53], and on expression of several vitamin D related pathways such as VDR, CYP24A1 and CYP27B1 [9, 54, 10] and HIF-1α [55]. These results are also in accordance with a hypothesis that active melanogenesis can promotes melanoma progression [56, 57, 52, 58], and with previous research showing that melanogenesis can increase immune-, chemo- and radioresistance of melanoma cells [59–64], and affects survival of melanoma patients after radiotherapy [65], which suggests that inhibition of melanogenesis can be considered as an adjuvant treatment in melanoma therapy [66, 67].

The reduced RORs expression in melanoma progression might promote tumor growth via multiple pathways, which are linked to the role of RORs in the regulation of circadian rhythm, glucose and lipid metabolism, or immune system (reviewed in [68]). For example, disturbance of circadian rhythm can promote cancer development [69], and a reduction of RORs expression promotes aerobic glycolysis and tumor cell growth (reviewed in [70, 68]). Finally, RORs, especially RORγ, are important regulators of inflammation and Th17 immune responses [68, 71]. Therefore, a reduced expression of RORs may affect a delicate balance between anti- and pro-tumorigenic actions of immune response.

Ligands for RORs, include several intermediates of the cholesterol biosynthetic pathway, cholesterol, and hydroxy derivatives and esters of cholesterol, which can act either as agonists or inverse agonists [7, 11–13]. Furthermore, previously we found that VDR expression decreased during progression of melanocytic lesions [9, 10] and that RORα and RORγ can act as receptors for novel vitamin D hydroxyderivatives [14]. Therefore, we suggest that disturbances in expression of RORα and RORγ in melanomas in combination with perturbations in skin endogenous metabolic pathways related to production of active forms of vitamin D or other secosteroids or sterols with their respective phenotypic actions could contribute to melanomagenesis and melanoma progression. This concept could be evaluated using appropriate animal models including patient-derived orthotopic xenografts, which mimic clinical tumor growth and metastasis [72, 73]. We also plan to perform an in depth mechanism oriented studies to define modes of action of RORs in melanomagenesis using patient-derived orthotopic xenografts and transgenic mice.

In conclusion, our studies indicate that development and progression of melanoma is associated with decreased expression of RORα and RORγ, and that downregulation of RORs level is associated with unfavorable clinical prognosis. These data indicate that expression and localization of RORs may serve as diagnostic tools and prognostic markers of the diseases outcome. We also suggest that targeting RORα and RORγ signaling may represent a promising strategy for melanoma treatment.

## MATERIALS AND METHODS

### Clinico-pathological studies

#### Tissue samples of human cutaneous melanocytic tumors

The patients were qualified into this retrospective study based on preliminary clinic-pathological data obtained from database of the Oncology Center in Bydgoszcz, Poland. Preliminary selection of patients was random and the criterion for the searching the electronic database of patients included the diagnosis of primary melanoma in the Oncology Center, followed by verification of paraffin formalin-fixed paraffin-embedded tissue blocks quality and the availability of tissue tumor in the section after routine diagnostic procedures. Normal skin samples [n=11] were obtained from patients who underwent surgery not related to skin diseases, while primary melanoma samples [n=79], melanoma metastases [n=39] and nevi [n=27] were excised from patients treated in the Oncology Centre in Bydgoszcz during the period 2003-2010 [54, 9]. Clinico-pathomorphological characterization of the patients included into this study is presented in Table 4. The mean follow-up time was 26.8 months (ranging from 1.4 to 84.0 months). Melanomas were classified AJCC Melanoma Staging and Classification [74, 75], briefly: pTis - melanoma in situ; pT1 – melanoma ≤ 1.00 mm in thickness, pT2 - 1.01-2.00 mm in thickness, pT3- 2.01-4.00 mm in thickness, pT4 - > 4.00 mm in thickness and N0 – no regional lymph node metastases detected, N1 - 1 metastatic lymph node presents, N2 – 2-3 metastatic lymph nodes present, N4 - 4+ metastatic nodes, or matted nodes, or in transit metastases/satellites with metastatic nodes. The study using human skin samples was approved by the Committee of Ethics of Scientific Research of Collegium Medicum of Nicolaus Copernicus University, Poland (approval number KB 448/2009).

**Table 4:** Patient and melanoma characteristics

Clinico-pathologic features	n
**Type of lesions**	
All samples	156
Melanocytic nevi	27
Primary melanomas	79
Nodular	39
Superficial spreading	38
Acral	2
Melanoma metastases	39
Normal skin	11
**Age (y)**	
Melanocytic nevi	Mean 40, median 35 (range 20-85)
Melanomas	Mean 61, median 59 (range 25-100)
**Male/female ratio**	
Melanocytic nevi	
M	7
F	20
Melanomas	
M	44
F	35
**Anatomical Site**	
Melanocytic nevi	
Extremities	6
Head and neck	5
Trunk	16
Melanomas	
Acral	2
Anogenital	3
Extremities	28
Head and neck	16
Trunk	30
**Breslow thickness (mm)**	
in situ	5
0-1	18
1.1-2	11
2.1-3	8
3.1-4	5
>4.0 0	32
**Clark level**	
I	10
II	7
III	23
IV	27
V	12
**pT**	
pT0	5
pT1	18
pT2	11
pT3	13
pT4	32
**pN**	
pN0	51
pN1	12
pN2	10
pN3	6
**pM (at the time of diagnosis/during follow-up)**	
pM0	79/70
pM1	0/9
**Overall stage (at the time of diagnosis/during follow-up)**	
0	5/5
1	21/20
2	24/17
3	29/28
4	0/9

M – males, F - females.

#### RORα and RORγ immunostaining

**Figure 14:**
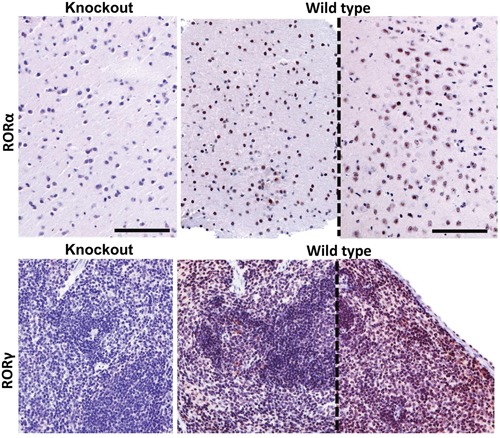
RORα (upper panel) and RORγ (lower panel) immunostaining of brain and spleen tissue sections of RORα or RORγ knockout mice and wild type mice (sections from two different mice, separated with dashed line) The mouse brain samples were stained with anti-RORα antibody and mouse spleen with anti-RORγ antibody. Scale bars - 100μm.

RORα and RORγ immunostaing was performed on standard formalin-fixed paraffin-embedded 4 μm sections of human samples, as described previously [14]. Briefly, after antigen retrieval sections were incubated overnight at 4°C with RORα or RORγ antibodies (respectively, goat anti-RORα antibody, clone C-16, Santa Cruz Biotechnology, Dallas, TX, dilution 1:25 and rabbit anti-RORγ antibody, generated and tested as previously described [14, 76], dilution 1:50. Then, sections were incubated for 30 min with secondary antibody (VECTASTAIN® Elite ABC Goat IgG, Vector Laboratories Inc., Burlingame, CA), followed by 30 min with ImmPACT™ NovaRED™ HRP substrate (Vector Laboratories Inc., Burlingame, CA) and visualisation with ImmPACT NovaRED (Vector Laboratories Inc., Burlingame, CA) for RORα immunostaining. For RORγ detection, sections were further incubated with secondary anti-rabbit antibody EnVision™ FLEX /HRP (Dako, Carpinteria, CA) and Vector NovaRED (Vector Laboratories Inc., Burlingame, CA). After counterstaining with hematoxylin, sections were dehydrated and mounted in permanent medium (Consul Mount; Thermo Fisher Scientific Inc. Waltham, MA, USA). Formalin-fixed paraffin embedded samples of brain and spleen of wild type and knockout mice were used for the assessment of the specificity of the RORα and RORγ antibodies, respectively (Figure 14). The RORγ KO mice (B6;129-Rorc<tm1Amj>) were described previously [77] and the RORα-deficient mice (RORasg/sg) were obtained from The Jackson Laboratory. Tissue sections were immunostained as described above with either the RORα antibody (1:500 dilution) or the RORγ antibody (1:250 dilution). Slides were either analyzed by Nikon Eclipse 80i light microscopy (equipped with Nikon Digital Sight DS Fi1-U2 digital camera and NIS-Elements BR 3.0 software (Nikon Instruments Europe BV, Badhoevedorp, The Netherlands).

#### Evaluation of RORα and RORγ immunostained sections

Immunostained sections of normal and pathological skin were analyzed without knowing the detailed histopathological diagnosis, malignancy grade and other clinical data. RORα and RORγ immunostaining intensity of melanomas was scored semiquantitatively, taking into consideration both staining intensity and percentage of immunostained tumor cells or, in normal skin, epidermal cells. The staining intensity was evaluated with reference to immunostaining of control, epidermal cells, scored as strong. The semiquantitative score was calculated as: SQ=mean (IR x SI), where IR is the percentage of immunoreactive cells and SI is the staining intensity from 0 to 3 arbitrary units (A.U.) with 0 as negative (0), weak (1), moderate (2) and strong (3). Then, cases were stratified according to RORs SQ-score as follows: SQ 0.0-10.0=none RORα, SQ 10.1-50.0=low RORα, SQ 50.1-300.0=high RORα, SQ 0.0-50.0=none RORγ, SQ 50.1-100.0=low RORγ, SQ 100.1-300.0=high RORγ.

Proliferative activity, melanin content evaluation and TILs were performed as previously described [53, 9, 54, 10, 78, 79].

#### Analysis of OS and DFS time

Clinico-pathological data related to the time between primary treatment (surgical excision) and histopathological diagnosis of melanoma to diagnosis of metastases were obtained from electronic database of the Oncology Center, Bydgoszcz Poland. Metastases were diagnosed by histopathological diagnosis of resected lesions, fine needle aspiration biopsy and/or diagnostic imaging techniques (magnetic resonance imaging, computed tomography). The date of deaths was obtained from the Department of Registry Office in Bydgoszcz, Poland. All data are presented according to REMARK guidelines [80].

### Cell culture-based study

#### Cells

Human SKMEL-188 and AbC1 hamster melanoma cells were cultured in Ham's F10 supplemented with 5% fetal bovine serum (FBS) and 1% antibiotics (penicillin/streptomycin/amphotericin, Sigma-Aldrich, St. Louis, MO) as described previously [51, 14]. Cells cultured in this medium remained amelanotic [51, 14]. Cells were seeded in chamber slides (for immunofluorescence study) or 60 mm dishes (for western blot study) in triplicate and after achieving 60% confluence, the media were change to the Ham's F10 medium supplemented with L-tyrosine (400 μM) to induce melanin pigmentation or Ham's F10 without addition (to maintain amelanotic phenotype) and after 4 days, pigmented and non-pigmented cultures were fixed in 4% paraformaldehyde or harvested by trypsinization for western blot analyses (WB). Human hepatoma (HEP) control cells overexpressing either RORα or RORγ were cultured as described previously [14] and processed for immunofluorescence or WB as above.

#### Fluorescent immunocytochemistry (F-ICC)

We have followed protocols described previously in [81, 14]. Briefly, permeabilization and blocking was performed with 0.2% Triton X-100 and 5% donkey serum in PBS for 1h, RT. Primary polyclonal antibody against goat RORα (#sc-6062, Santa Cruz Biotech, Santa Cruz, CA) and rabbit RORγ (developed at NIEHS [14, 76]) diluted in the same blocking solution were applied overnight at 4°C. After extensive rinsing in PBS, the secondary species specific antibody [donkey anti goat Igγ conjugated Alexa488 (green) or donkey anti rabbit Igγ conjugated Alexa594 (red); both from Santa Cruz] were applied for 1 h, RT. The positive control consisted of hepatoma cells (HEPA) overexpressing either RORα or RORγ, the negative control constituted the same F-ICC procedure except of omitting of the primary antibody. After extensive rinsing the slides were topped with mounting media allowing for nucleus visualization with either propidium iodie (PI, red) or 4′,6-diamidino-2-phenylindole (DAPI, blue) and examined under fluorescent microscope (Leica, Digital DM4000B, Buffalo Grove, IL) equipped with appropriate filters capable for visualization of green, red and blue fluorophores and conjugated to a digital camera. Pictures were further analyzed for intensity of immunocomplexes with the use of ImageJ software (National Health Institute, Bethesda, MD).

#### Western blot (WB)

Detailed description of WB method is provided in [14]. Shortly, harvested cells were subjected to nuclear and cytoplasmic protein isolation with Nuclear Extraction Kit (#400100, Active Motif, Carlsband, CA) according to the manufacturer instructions. Equal amounts (30 μg) of protein were denatured with Laemli buffer, subjected to SDS/PAGE and transferred to a PVDF membranes and incubated with antibodies [rabbit RORα (#GTX100029, GeneTex, Irvine, CA); rabbit RORγ (see above); rabbit Lamin A (#sc20681, Santa Cruz); and mouse monoclonal β-actin conjugated-HRP (#A3854, Sigma, Saint Louis, MO). After extensive washing in TBS-T the membranes were incubated with secondary species specific Igγ conjugated-HRP for 2 h, RT and detection of immunocomplexes was performed with chemiluminescence. Calculation of normalized expression levels of RORs antigens was achieved by dividing the mean signal intensity (density) of expressed proteins (evaluated with ImageJ, NIH) with the internal (Lamin A, for nuclear samples) and loading (β-actin) controls. Next, we subtracted the values from amelanotic out of melanized samples and provided them as %.

### Statistical analyses

Statistical analysis was performed with the Prism 5.00 (GraphPad Software, San Diego, CA). Results were considered as statistically significant when P < 0.05. For comparison two groups the Student's *t*-test were used, and for comparison three or more groups – ANOVA. To evaluate the association between RORα and RORγ staining and categorical variables, the Pearson's correlation was used. Survival analysis was performed using Log-rank test. For analysis of survival after adjustment of Breslow thickness and overall stage the Cox proportional-hazards regression analysis was performed using NCSS10 (NCSS, LLC, Kaysville, Utah, USA). Data are presented as mean ±SD.
